# The association of a team management model based on Timing It Right theory with outcomes after cesarean section

**DOI:** 10.1097/MD.0000000000050872

**Published:** 2026-10-02

**Authors:** Xiaoya Wang, Yifeng Zhang, Shengnan Ren, Yan Zhao

**Affiliations:** a Shandong Provincial Hospital Affiliated to Shandong First Medical University, Jinan, Shandong, China.

**Keywords:** cesarean section, discharge readiness, team management, Timing It Right

## Abstract

To evaluate whether a team management model based on Timing It Right theory was associated with postoperative recovery, breastfeeding outcomes, and readiness for hospital discharge among women undergoing elective cesarean section. This single-center, nonrandomized quasi-experimental study used a sequential (nonconcurrent) control design. Women undergoing elective cesarean section were recruited by convenience sampling. Participants treated from April to June 2023 received routine perioperative clinical pathway management and served as the control group, whereas participants treated from July to September 2023 received the same routine pathway plus a team management intervention based on Timing It Right theory. Outcomes related to postoperative recovery, lactation, breastfeeding self-efficacy, and readiness for hospital discharge were compared between groups at prespecified time points. A total of 97 cases were studied in the 2 groups, including 49 cases in the control group and 48 cases in the intervention group. Comparing the postoperative clinical data of the 2 groups, the number of active steps and the time of the first urination in the intervention group were better than those of the control group in the postoperative period of 24 and 48 hours (*P* < .05); comparing the postoperative lactation situation of the 2 groups, the amount of lactation in the intervention group was better than that in the control group in the postoperative period of 48 and 72 hours (*P* < .05), and breastfeeding confidence in the intervention group (126.65 ± 15.894) was better than that of the control group (110.13 ± 17.662), and the difference between the 2 groups was statistically significant (*P* < .05). In addition, the total discharge readiness score of the intervention group (98.55 ± 12.261) and adaptability dimension (39.65 ± 6.851), anticipatory support dimension (26.06 ± 3.875) were better than that of the control group (82.46 ± 15.276, 33.06 ± 6.166, 20.48 ± 3.968), and the difference between the 2 groups was statistically significant (*P* < .05). In this single-center, nonrandomized sequentially controlled study, the team management model based on Timing It Right theory was associated with better early postoperative activity, earlier first urination, more favorable lactation status at 48 and 72 hours, higher breastfeeding self-efficacy, and greater readiness for hospital discharge.

## 1. Introduction

Maternal and child health is the cornerstone of a country’s and society’s development, and the Global Strategy for Women’s, Children’s and Adolescents’ Health (2016–2030) clearly states that 52% of maternal deaths can be avoided by strengthening maternal management.^[1]^ A cesarean section is a surgical operation to remove the fetus and its appendages through an abdominal incision of the uterus when the gestation cycle is 28 weeks and above. When a pregnant woman has a high-risk pregnancy or difficult labor, a timely cesarean section is of great significance to ensure the safety of the mother and the baby. A survey shows^[2,3]^ that the cesarean section rate in China in 2020 was 43.79%, significantly higher than the world average cesarean section rate of 21.1%.^[4]^ With the implementation of China’s fertility policy, the number of women in labor has changed, and the new demographic characteristics have also impacted the traditional obstetrics medical service model.^[5]^ While changes in maternal demand, the balance between the “supply” of healthcare institutions and the “demand” of pregnant women has become an important gap in the clinical. Discharge readiness^[6]^ is the patient’s perception and evaluation of whether they are ready to be discharged from the hospital, to a certain extent, to respond to the patient’s in-hospital physical and psychological recovery, but also to a certain extent, to predict the degree of safety of the patient’s post-discharge home care, a large number of research results show that^[2,7,8]^ China’s maternal readiness for discharge is low, and the analysis of the reasons for this is mainly related to the shortening of the length of hospitalization, the mother’s short time of adaptation, the low degree of adaptation to role change, and the surgical procedure. Some studies have shown that poor experience in the early postoperative period after cesarean section is an independent risk factor for the occurrence of postpartum depression,^[9]^ so good readiness for discharge is a prerequisite for cesarean section mothers to be discharged from the hospital.

Secondary caregiver is mainly engaged in medical care. As an auxiliary force for nursing services, a secondary caregiver can play an important role in life care services. Research shows that foreign developed countries already have more mature secondary caregiver career training programs, standardized secondary caregiver service objects and service scopes, and provide social security for this purpose. In 2015, the number of medical secondary caregiver in the United States reached 1.42 million, and the survey data of some countries in Europe show that the number of secondary caregivers exceeds 15 per 1000 population and is on a rising trend year by year. In 2023, the government issued the “Action Plan for Further Improvement of Nursing Services (2023–2025)” policy requires strengthening the standardized management of secondary caregiver, with special emphasis on elderly care and maternal and child care, and the entry of secondary caregivers who care for mothers and infants into healthcare institutions has become an inevitable trend, so this study introduces secondary caregivers who care for mothers and infants into the management system of obstetrics wards. 2008 Cameron and Gignac proposed the Timing It Right theory,^[10-13]^ which divides the disease into diagnostic, stabilizing, preparatory, adjusting, and adaptive phases, and emphasizes the implementation of dynamic and continuous interventions for the individual at different stages of the disease. This model has been effectively applied in patients with myocardial infarction and other patients. This study analyzes the effect of team management based on Timing It Right theory on discharge preparation services for cesarean section mothers.

## 2. Materials and methods

### 2.1. Study design and participants

This was a single-center, nonrandomized quasi-experimental study with a sequential (nonconcurrent) control group. Women undergoing elective cesarean section and hospitalized in the obstetrics ward of Shandong Provincial Hospital Affiliated to Shandong First Medical University were recruited by convenience sampling. Inclusion criteria were elective cesarean section, clear consciousness and ability to communicate, written informed consent, voluntary participation, and ability to complete the intervention. Exclusion criteria were emergency surgery or need for a second operation; cardiac, hepatic, renal, or pulmonary organic dysfunction; transfer of the newborn to another department; or refusal to breastfeed. Participants who withdrew for any reason were considered dropouts. The sample-size calculation was based on a 2-sided comparison of 2 independent means (mean difference from previous studies, 2.39; standard deviation, 3.24; α = 0.05; power = 0.90) and allowed for a 20% dropout rate, yielding a target of at least 48 participants per group. A nonconcurrent control design was used: women recruited from April to June 2023 constituted the control group, and women recruited from July to September 2023 constituted the intervention group. Medication and rehabilitation exercises were intended to be comparable between groups. Because the groups were enrolled in different calendar periods rather than randomized concurrently, temporal changes in clinical practice, staffing, seasonal factors, or unmeasured patient characteristics could not be fully excluded as potential confounders. The study was approved by the Ethics Committee of Shandong Provincial Hospital Affiliated to Shandong First Medical University (SWYX:NO.2023-393), and all participants provided written informed consent.

### 2.2. Methodologies

The control group adopted routine clinical pathway management. The specific content included admission health education: introducing the hospital ward’s environment and relevant management system to pregnant women and their caregivers, and carrying out relevant medical and nursing assessments; perioperative management: doctors and nurses explained the relevant contents of the preoperative and postoperative period, informed them of the relevant precautions, and instructed the mothers to carry out breastfeeding after the operation. Discharge guidance: informing the mother and her caregivers of the precautions to be taken outside the hospital, such as rational use of medication, rehabilitation exercises, diet, and exercise, and telling them to repeat the examination on time.

The intervention group implemented team management based on Timing It Right theory in addition of routine clinical pathway management in the control group. Management team formation. The management team included doctors, nurses, and secondary caregiver who care for mothers and infants, all of whom were on staff in the obstetrics ward of the hospital. Formation of the First Draft of the Intervention Program. A. Theoretical Basis: The intervention was based on Timing It Right theory, which, also known as “timely intervention theory,” divides the course of the disease into 5 periods, emphasizing that the patient’s needs change with the different stages of the disease. In this study, the pregnant women who were to undergo elective cesarean section were divided into 5 periods: the diagnostic period, from the time of the pregnant woman’s admission to the hospital until she was determined to undergo a cesarean section; the stabilization period, from the time of determining a cesarean section to 1 day prior to the operation. The preparation period is the preoperative phase on the day of surgery; the adjustment period is the postoperative period from the day of surgery to 1 day postoperatively; and the adaptation period is from 1 day postoperatively to just before discharge from the hospital. The 5 phases may overlap with each other because patients’ preoperative waiting time varies. B. Literature retrieval: “cesarean section” combined with “discharge” “discharge preparation program” “discharge preparation” “Discharge plan” “Discharge readiness” as Chinese search terms, with “Puerpera OR Cesarean section OR Obstetrical department OR Pregnant woman AND Discharge OR Readiness for hospital discharge OR RHD OR Ready to discharge OR Discharge plan” as the English search terms, and searched the CNKI (China National Knowledge Infrastructure), Wanfang, and VIP (VIP information), Sinomed, PubMed, Web of Science, Embase and other foreign databases were searched from the establishment of the database to January 31, 2023. The obtained literature was organized and useful information was summarized. Finally, group discussion was conducted to form the first draft of the intervention program for cesarean section maternal discharge preparation services, with doctors, nurses, and secondary caregivers who care for mothers and infants as the horizontal level 1 indicators. Each horizontal level 1 program included 3 secondary indicators for maternal, newborn, and family members, with 5 time nodes as the vertical axis, namely, admission, preoperative period, the day of the operation, 1 day after the operation, and 2 days after the operation–discharge, and at different time nodes, different team service providers provide different contents to achieve the purpose of promoting health and improving the coping efficacy of mothers and their families. Expert Correspondence and Formation of the Final Draft. A. Expert Selection: 16 experts from 7 hospitals were selected for Delphi expert consultation in this study, and the inclusion criteria for the experts were: engaged in obstetrics clinical medical care, nursing care, and maternal and infant rehabilitation, with a deeper understanding of the field; undergraduate education or above; and intermediate or above title. B. Expert Correspondence Questionnaire: Includes 4 parts: Preamble: Includes the background of the study, the purpose, significance, and requirements for completion; the main body of the correspondence questionnaire: including the intervention program, connotation, time and mode, etc, and the importance of the intervention content was scored using the Likert 5-level scoring method, which was very important, important, general, unimportant, and unimportant respectively, and assigned a score of 1–5; basic information of the experts: including the names, genders, titles, job titles, working years, work areas, and judgmental basis for this study and familiarity, and degree of familiarity. C. Implementation of Expert Consultation: 2 rounds of expert consultation were conducted by the researcher in February 2023 in the form of distributing and recycling the correspondence questionnaires via email or WeChat. After the first round of questionnaires were collected, the research team collated, summarized, and analyzed the experts’ opinions, modified or deleted the corresponding entries, and the criteria for inclusion of entries were that the importance score was >3.5 and the coefficient of variation (CV) was <0.25, so as to form the second round of questionnaires with a summary of the opinions of the experts’ consultations in the first round. In the 2 rounds of expert consultations, 16 questionnaires were sent out in round 1, 15 questionnaires were collected, and the experts’ positive coefficient was <0.25, and the experts’ positive coefficient was <0.25. In the 2 rounds of expert correspondence, 16 questionnaires were sent out in the 1st round, 15 questionnaires were recovered, and the positive coefficient of experts was 93.75%; 16 questionnaires were sent out and 16 questionnaires were recovered, and the positive coefficient of experts was 100.00%, which indicated a higher degree of enthusiasm among experts. After 2 rounds of correspondence, the experts’ opinions showed a good tendency to concentrate and the correspondence was stopped. The judgmental basis of the 2 rounds of correspondence was 0.97, the familiarity (Cs) was 0.93, the coefficient of expert authority (Cr) was 0.95, and expert Cr > 0.70 was acceptable reliability, indicating a high degree of expert authority in this study. The team further modified and improved the intervention program and determined the formal version after collective review. Team management: the nurse manager is mainly responsible for and organizes weekly group meetings with members, which include questions and answers during team implementation, the latest obstetrics theories, and clinical practice management norms, etc, to ensure that the team’s work is carried out smoothly.

Intervention delivery and interaction schedule. The intervention was organized around 5 prespecified perioperative time points: admission (diagnostic phase), the preoperative period (stabilization phase), the day of surgery (preparation phase), postoperative day 1 (adjustment phase), and postoperative day 2 through discharge (adaptation phase). At these time points, physicians, nurses, and maternal-infant secondary caregivers delivered role-specific support during routine clinical encounters, with content tailored to the woman’s and family’s needs. Core content included medical assessment and symptom management; perioperative and mobilization education; urinary catheter removal and early activity guidance; breastfeeding education, lactation stimulation, and hands-on breastfeeding assistance; assistance with maternal daily activities; newborn care skills; and family education for continued care after discharge. Problems identified by secondary caregivers were fed back to nurses and physicians for timely management, and the nurse manager convened weekly team meetings to review implementation questions and current obstetric practice. The protocol specified the timing, responsible team member(s), and core content of contacts but did not prescribe a fixed duration in minutes for each encounter; contact duration was individualized to clinical needs, and exact encounter duration was not prospectively recorded.

### 2.3. Evaluation tools

1. The Readiness for Hospital Discharge Scale (RHDS) is used to assess patients’ readiness for hospital discharge and consists of 12 items in 3 dimensions: personal status, adaptability and anticipatory support. Each entry is scored from 0 to 10, with 0 representing “not at all ready” and 10 representing “completely ready.” Higher discharge readiness scores indicate that the patient is better prepared or more prepared for hospital discharge. The Cronbach α coefficient of the scale is 0.89 and the content validity index is 0.88.^[14]^.
2. Breastfeeding Self-Efficacy Scale (BSES): including 3 dimensions of feeding knowledge, feeding emotion, and feeding skills, using a scale of 1 to 5. The higher the score, the stronger the self-confidence. Cronbach α is 0.867,^[15]^ the scale has good reliability and validity.
3. lactation Maternal lactation was collected daily by a stationary nurse, using the 30-seconds manual milking method^[16]^ to estimate the amount of lactation, no milk as no milk or only 1 to 2 drops of milk were squeezed out; little milk as the mother could slowly secrete milk when squeezed by hand, 0.5 to 1 mL in 30 seconds; medium milk as the milk could be continuously secreted when squeezed by hand, and it could be up to 2 mL or so in 30 seconds; and much milk as a large amount of milk could be secreted in large quantities or in jets when squeezed by hand, and more than 2 mL was secreted in 30 seconds. The milk was collected every day by a stationary nurse, and it was estimated using the 30-s manual milking method. The milk is secreted in large quantities or in jets when squeezed by hand, exceeding 2 mL in 30 seconds.
4. Steps out of bed: Both groups were issued smart exercise bracelet recorders to record the number of steps walked per day.

### 2.4. Data collection

Maternal general and specialized conditions were assessed on admission and immediately after returning to the ward. Lactation was collected at 24, 48, and 72 hours postoperatively. Maternal readiness for discharge and breastfeeding self-confidence were measured at discharge. The questionnaires were completed and returned on the spot using a standardized instruction manual, and the researchers responded objectively to any questions. All data were counted, checked, entered, and logically corrected by 2 trained researchers simultaneously to reduce assessment subjectivity and error in the results.

### 2.5. Data processing

All the data were double-checked, entered, and analyzed statistically by SPSS24.0 statistical analysis software. Measurement data were tested for normality by “One-Sample Kolmogorov Smirnov Test,” and for homogeneity of variance by “Homogeneity of Variance Test.” “Homogeneity of Variance Test” was used for the test of normality, and the mean ± standard deviation (*x* ± *s*) was used to express the continuous data that conformed to normal distribution, and the median (*M*) and quartile (*Q*) were used to express those that did not conform to a normal distribution. Categorical data were described by frequency and percentage (%). When comparing the research indexes of the 2 groups of research subjects, a 2-independent-samples *t* test was chosen after exploratory analysis, and a nonparametric Wilcoxon rank-sum test was chosen for the data that did not meet the normal distribution. The difference was considered statistically significant at *P* < .05.

## 3. Results

### 3.1. Comparison of the general data of the 2 groups of patients

The 2 groups of patients finally collected complete information on 97 cases, including 48 cases in the control group and 49 cases in the intervention group. The difference between the 2 groups in general information was not statistically significant (*P* > .05), as shown in Table 1.

**Table 1:** General information (n = 97).

		Intervention group	Control group	*t/x^2^*	*P*
	Age	31.35 ± 4.94	31.71 ± 4.96	-0.359	.720
	Length of pregnancy (d)	270.45 ± 6.32	271.79 ± 7.47	-0.956	.341
Careers	Employees	20	13	3.970	.410
	Self-employment	14	14		
	Peasant	8	7		
	Not have	2	5		
	Other else	5	9		
Educational attainment	Junior high school and below	4	10	3.263	.196
	High school and junior college	15	14		
	Undergraduate and above	30	24		
Monthly salary	<5000	17	24	3.784	.286
	5000–10,000	12	14		
	10,000–15,000	14	7		
	>15,000	5	4		
Primary caregiver	Mate	17	17	0.702	.873
	Ascendant	17	16		
	Other	12	10		
	All 3	3	5		
Number of deliveries	0–2	19	21	8.359	.213
	>2	30	27		
Number of surviving children	<2	23	21	1.519	.678
	≥2	26	27		
Pregnancy-related complications	Not have	31	37	2.209	.137
	There are	18	11		
Complications of pregnancy	Not have	44	42	3.033	.082
	There are	5	6		

* Pregnancy complications: uterine fibroids in pregnancy, hypothyroidism in pregnancy, anemia in pregnancy, diabetes mellitus in pregnancy, liver function abnormalities in pregnancy, etc.; pregnancy complications: excessive/poor amniotic fluid, placenta praevia, hypertension in pregnancy, placenta previa, etc.

### 3.2. Comparison of clinical data between the 2 groups of patients

The intraoperative conditions, postoperative complications, and uterine blood accumulation were compared between the 2 groups, and the difference between the 2 groups was not statistically significant (*P* > .05), as shown in Table 2.

**Table 2:** **Clinical information (n = 97**).

		Intervention group	Control group	*t/x^2^*	*P*
Clinical information	Intraoperative bleeding	289.58 ± 95.069	294.48 ± 90.460	-0.200	.842
	Neonatal scoring	9.92 ± 0.238	9.94 ± 0.320	-1.424	.107
	Newborn weight	3393.33 ± 378.26	3278.44 ± 435.7	1.379	.171

### 3.3. Comparison of postoperative data between the 2 groups of patients

Postoperative outcomes were compared between groups. At 24 and 48 hours after surgery, the intervention group recorded more active steps, and the time to first urination was shorter than in the control group (*P* < .05), as shown in Table 3.

**Table 3:** Comparison of postoperative data (n = 97).

	Sports event	Intervention group	Control group	*t/x^2^*	*P*
First time (h)	Getting out of bed	23.115 ± 3.996	23.958 ± 5.219	-0.889	.376
	Ventilate	31.052 ± 9.260	35.104 ± 10.765	-1.611	.114
	Pass water	23.375 ± 3.418	26.646 ± 5.200	-2.843	.005
Steps out of bed	24 h after surgery	89.81 ± 44.083	65.00 ± 38.029	3.087	.003
	48 h after surgery	220.90 ± 60.790	136.88 ± 56.544	4.817	.001
Postoperative complication	Yes	2	3	0.211	.646
	No	47	45		
Uterine blood	Yes	2	2	0.201	.620
	No	47	46		

### 3.4. Comparison of postoperative lactation in 2 groups of patients

Lactation status was compared at 24, 48, and 72 hours after surgery. No statistically significant between-group difference was observed at 24 hours, whereas lactation status was more favorable in the intervention group at 48 and 72 hours (*P* < .05), as shown in Table 4.

**Table 4:** Comparison of lactation in postpartum period.

		Intervention group	Control group	*x^2^*	*P*
24 h	No	23	24	0.190	.910
	Little	23	23		
	Medium	3	2		
	A lot	0	0		
48 h	No	3	2	12.229	.007
	Little	21	36		
	Medium	20	9		
	A lot	5	1		
72 h	No	1	1	26.812	.001
	Little	2	19		
	Medium	21	19		
	A lot	25	9		

### 3.5. Comparison of breastfeeding confidence between the 2 groups

Comparison of breastfeeding confidence between the 2 groups showed that the intervention group was better than the control group in all dimensions of breastfeeding confidence and total score (*P* < .05), as shown in Table 5.

**Table 5:** **Comparison of breastfeeding confidence**.

	Intervention group	Control group	*t*	*P*
Skill dimension	30.12 ± 3.914	25.10 ± 6.082	23.450	<.001
Emotional dimension	74.71 ± 6.175	60.24 ± 9.457	79.696	<.001
Cognitive dimension	25.61 ± 3.679	20.94 ± 3.872	37.166	<.001
Total breastfeeding self-confidence score	126.65 ± 15.894	110.13 ± 17.662	23.492	<.001

### 3.6. Comparison of discharge readiness between the 2 groups

The readiness for discharge was compared between the 2 groups, and it was found that the intervention group was better than the control group in terms of adaptability, anticipatory support dimensions, and total score (*P* < .05), as shown in Table 6.

**Table 6:** **Comparison of discharge readiness of patients in 2 groups**.

	Intervention group	Control group	*t*	*P*
Personal status dimension	23.63 ± 2.667	21.73 ± 4.916	1.682	.198
Adaptive capacity dimension	39.65 ± 6.851	33.06 ± 6.166	24.766	<.001
Anticipatory support dimension	26.06 ± 3.875	20.48 ± 3.968	49.135	<.001
Total discharge readiness score	98.55 ± 12.261	82.46 ± 15.276	32.806	<.001

## 4. Discussion

### 4.1. Team management based on Timing It Right theory was associated with early postoperative recovery indicators

Cesarean section mothers face a series of stimuli such as perioperative fasting, intraoperative catheter stimulation, postoperative incision pain, and contraction pain. The number of steps out of bed and the time of the first urination are important indicators to reflect their early postoperative recovery. The results of the study showed that the number of steps out of bed in the intervention group was higher than that in the control group 24 and 48 hours after the operation, and the time of the first postoperative urination was earlier than that in the control group. These between-group differences are consistent with a possible beneficial association between the management model and early postoperative recovery; however, the nonrandomized sequential design does not permit a causal conclusion. Analyzing the reasons, puerpera after cesarean delivery face the double pressure stimulation of physical and psychological role change. At present, most of them are mothers and infants in the same room, facing multiple pressures such as environmental changes and sleep time disorders. The sense of maternal adverse experience rises, the physical load is large, and the puerpera and newborn need adequate life care. However, the caregivers are mostly the spouses or the elders of the family, and most of them suffer from the deficiencies of insufficient life care and other deficiencies. Healthcare workers can ensure adequate medical care and safety for puerpera and babies, but they are not capable of meeting the needs of puerpera and babies at any given time, and there is a gap between the “supply” of healthcare workers and the “demand” of mothers and their families. Therefore, this study incorporates secondary caregivers who care for mothers and infants into the management team of obstetrics wards, strictly controls the incorporation, assessment, and work process of secondary caregivers who care for mothers and infants, standardizes the concept of mother and baby care and related nursing measures, and emphasizes the cultivation of humanistic care. At the same time, the team meets the different needs of the patients according to their different stages. For example, the first day after the operation to face a variety of physical discomfort, etc, this study regarded it as a period of adjustment, such as the doctor to give the necessary pain medication, nurses to give the removal of urinary catheters and the necessity of getting out of bed activities of the mission, mother and baby caregivers to give physical assistance, etc, to meet their needs in a timely manner. The results showed that the indicators related to postoperative rehabilitation of cesarean section mothers under this management model were better than those of the control group, suggesting that this model may have clinical value. Nevertheless, the present study was not designed to determine whether the model itself caused these differences or whether it relieves the work pressure of healthcare personnel, but also improves the difficulties of mothers and their families with insufficient maternal and infant care.

### 4.2. Team management based on Timing It Right theory was associated with lactation and breastfeeding self-efficacy

The World Health Organization has advocated that the optimal form of feeding for newborns in the first 6 months of life is breastfeeding. In this study, the lactation and breastfeeding confidence of cesarean section mothers were more favorable in the group receiving team management based on Timing It Right theory than in the control group. It has been pointed out that the main reason for the high rate of cesarean section in China is the presence of relevant maternal comorbidities or obstructed labor, which are also important factors affecting breastfeeding.^[17,18]^ Cesarean section mothers have reduced breastfeeding efficacy due to lack of breastfeeding knowledge and skills, anesthesia factors, and incision pain. Based on this, in this study, the team dynamically gave them stage-by-stage interventions, such as on the first postoperative day, the medical staff stimulated lactation through breast acupoints and informed them of the ways to maintain lactation and the importance of lactation. Secondary caregivers who care for mothers and infants assisted mothers in breastfeeding, encouraged mothers and their families to adhere to breastfeeding, and subconsciously conveyed the knowledge of breastfeeding to the mothers and their families. At the same time, the secondary caregivers who care for mothers and infants provided feedback to the medical staff about problems in the process of breastfeeding, so as to solve the problems in the process of lactation and breastfeeding in a timely manner. The results showed that there was no statistically significant difference in lactation between the 2 groups in the 24-hour postpartum period, but after 48 and 72 hours, the lactation of the intervention group was significantly better than that of the control group, and the self-confidence in breastfeeding was significantly higher than that of the control group. Given the sequential, nonrandomized allocation, these findings should be interpreted as an association and not as proof that the intervention caused the observed differences in lactation or breastfeeding self-efficacy.

### 4.3. Team management based on Timing It Right theory was associated with readiness for hospital discharge

Discharge readiness reflects the physical and psychological recovery of mothers in the hospital, and also predicts, to some extent, the safety of patients’ home care after discharge, and the anticipatory judgment of personal coping and family social support. The results of this study showed that the discharge readiness of mothers who practiced the team management model based on Timing It Right theory was higher than that of the control group, and the adaptive ability and anticipatory support dimensions were significantly higher than those of the control group. Most of the mothers in this study chose to spend the puerperal period at home after discharge from the hospital, and the reasons for the difference in discharge readiness may be related to the mastery of personal skills and family support. During the hospitalization period, the team paid attention to the different stages of maternal and neonatal care after cesarean section, and instilled obstetric concepts into the mothers and their families. At the same time, the team seized the optimal period for the mothers and their families to adapt to the role of caregiving and to master caregiving skills. During the skills of caregiving, and in the period of adaptation, instructed on how to correctly guide mothers in daily life aspects such as turning over, scrubbing the body, and replacing and arranging personal objects, as well as how to correctly change newborns’ living objects. During the period of adaptation, family members are instructed on how to correctly instruct mothers to turn over, scrub their bodies, change and organize their personal belongings, and how to correctly change the newborn baby’s daily necessities, etc. They are also emphasized on the relevant points of care, such as how to correctly identify the hunger of newborn babies, and how to correctly assist mothers in placing themselves in the position of breastfeeding, and are guided to show care for the mothers and newborn babies in action in their work. Therefore, sufficient medical and nursing resources in the hospital, and correct maternal and infant care concepts and skills received by family members, may provide a basis for out-of-hospital care and may contribute to maternal adaptability and anticipatory support. The results of this study show that the intervention group’s readiness for discharge is higher than that of the control group, which supports the feasibility of further evaluating maternal-infant caregivers as part of team-based obstetric management. Because this study used a nonrandomized, nonconcurrent control group, the observed difference cannot be attributed solely to the intervention.

### 4.4. Strengths and limitations

This study has several strengths, including a multidisciplinary team intervention grounded in Timing It Right theory, a structured expert-consultation process, use of validated instruments (RHDS and BSES), and assessment of several clinically relevant perioperative outcomes. However, the findings should be interpreted in light of important limitations. First, this was a single-center, nonrandomized quasi-experimental study using a sequential (nonconcurrent) control group: control participants were recruited from April to June 2023 and intervention participants from July to September 2023. Therefore, secular changes in clinical routines, staffing, seasonal conditions, patient case mix, or other unmeasured factors may have confounded the between-group differences, even though measured baseline characteristics were similar. Second, the final analysis included only 97 participants from 1 institution, which limits statistical precision and external generalizability to hospitals with different cesarean-section pathways, staffing models, caregiver resources, and patient populations. Third, the intervention was delivered within routine clinical care, and contact duration was individualized rather than prospectively standardized or recorded, which limits assessment of intervention dose and reproducibility. Fourth, the study focused on short-term in-hospital outcomes and did not evaluate whether differences in breastfeeding, functional recovery, or discharge readiness persisted after discharge. Larger, multicenter, prospectively designed studies with concurrent allocation, standardized intervention-fidelity measures, and longer follow-up are needed to confirm these associations.

In this single-center nonrandomized quasi-experimental study with a sequential control group, team management based on Timing It Right theory was associated with more favorable indicators of early postoperative recovery, lactation, breastfeeding self-efficacy, and readiness for hospital discharge after elective cesarean section. The model may provide a useful framework for coordinating physicians, nurses, maternal-infant secondary caregivers, mothers, and family members across perioperative stages. However, the small single-center sample, nonconcurrent group recruitment, potential temporal confounding, lack of standardized contact-duration data, and short follow-up preclude causal inference and limit generalizability. Multicenter prospective studies with concurrent comparison groups and standardized fidelity assessment are warranted.

## 5. Conclusion

It is the responsibility of medical institutions, the requirement of society, and the demand of mothers and their families to promote the high-quality development of maternal and child safety and further improve the level of maternal and child health services. This study constructed a team management program through the Delphi method with Timing It Right theory as the theoretical basis, and proved the scientificity and feasibility of this management model through clinical research, which provides a certain reference basis for the perioperative management of cesarean section mothers. However, the results of this study were only confirmed in 1 medical institution, and the sample size can be expanded, and a multicenter study can be conducted at a later stage to further validate the application effect of this model.

## Author contributions

**Conceptualization:** Xiaoya Wang, Yifeng Zhang, Shengnan Ren, Yan Zhao.

**Data curation:** Xiaoya Wang, Yifeng Zhang, Shengnan Ren, Yan Zhao.

**Formal analysis:** Xiaoya Wang, Yifeng Zhang, Shengnan Ren, Yan Zhao.

**Funding acquisition:** Yan Zhao.

**Investigation:** Yan Zhao.

**Writing – original draft:** Yan Zhao.

**Writing – review & editing:** Yan Zhao.
